# Imaging in Healthcare: A Glance at the Present and a Glimpse Into the Future

**DOI:** 10.7759/cureus.36111

**Published:** 2023-03-14

**Authors:** Eman Nabrawi, Abdullah T Alanazi

**Affiliations:** 1 Department of Health Informatics, King Saud bin Abdulaziz University for Health Sciences (KSAU-HS), Riyadh, SAU; 2 Department of Health Informatics, College of Public Health and Health Informatics, King Saud bin Abdul-Aziz University for Health Science (KSAU-HS), Riyadh, SAU; 3 Department of Health Sciences, King Abdullah International Medical Research Center (KAIMRC), Riyadh, SAU

**Keywords:** vendor neutral archive (vna), pacs, image mining, image in healthcare, image informatics

## Abstract

The utilization of artificial intelligence (AI) applications in medical imaging relies heavily on imaging informatics. That is a one-of-a-kind professional who works at the crossroads of clinical radiography, data science, and information technology. Imaging informaticians are becoming crucial players in expanding, assessing, and implementing AI in the medical setting. Teleradiology will continue to be a cost-effective healthcare facility that expands. Vendor neutral archive (VNA) isolates image presentation and storing systems, permitting platforms to develop quickly, and is a repository for organization-wide healthcare image data. Efforts are made to incorporate and integrate diagnostic facilities such as radiography and pathology to fulfill the needs and demands of targeted therapy. Developments in computer-aided medical object identification may alter the environment of patient services. Finally, interpreting and processing distinct complex healthcare data will create a data-rich context where evidence-based care and performance development may be driven.

## Introduction and background

Advancement in technology has affected healthcare services, and the revolution in imaging informatics is spectacular. The changes were rapidly changing the lives of radiologists significantly. How was the imaging department decades ago, what is happening now, and more importantly, what will happen soon? [1]. This paper aims to provide an overview of the current image technology in healthcare and to explore emerging technology and applications by reviewing the most recent literature. Imaging is a unique medical subsidiary with distinct technical challenges [2]. Imaging is a branch of medicine that applies advanced technologies to diagnose and treat diseases. Imaging has several modalities, each with unique features, mechanisms, and techniques. Diagnostic radiology permits physicians to view the body's parts [3]. Diagnostic radiologists are physicians who specialize in the assessment of these images. The radiologist or other physician can determine the source of concerns, track how the body reacts to the medication, and assess other ailments, such as cancer and heart disease. A conventional X-ray is a form of electromagnetic energy that penetrates objects to reflect an image capable of representing the internal structure within the intended objects. It is influential in diagnosing and treating diseases. For example, a conventional radiograph displays variations among bones, air, and occasionally fat, making it helpful in assessing bone problems and chest diseases. Contrast agents must be used to compensate for the lack of conventional contrast among neighboring structures of equal radiography density. In traditional radiography, the patient is positioned within an X-ray tube and a film or detector responsive to X-rays. The contrast and spatial resolutions are affected by the selected film and the intensifying screen (which does not directly expose the film). Substances are needed to handle the film and are a common cause of faults and redo. Post-radiation exposure results in a fixed image that is hard to alter. Fluoroscopic screens, movies, and computer monitors can all be used to view the visuals. As the focal point of the X-ray tube, X-rays appear as a deviating conical beam. As a result, the radiography projection yields different results [4]. Unlike X-ray, ultrasound does not produce ionizing radiation. Traditionally, a sonographer or radiologist uses gel on the patient's skin and glides a transducer across the patient's body. The gel generates an acoustic bridge between the transducer and the skin, allowing an improved soundwaves transfer and thus producing images. The transducer transmits and receives high-frequency sound waves that penetrate and reflect the human body's internal structures. The intensity of the reflected sound waves and the time it takes to return is classified. The time it takes for the echo to reflect from its encounter with an acoustic edge, a structure inside the body that indicates sound, permits its position and location in the image to be allocated [5].

Ionizing radiation is used in computed tomography "CT" to obtain an axial image. CT enables viewing a broader range of tissue structures than the four essential concentrations noticeable in a traditional image (air, bone, soft tissue, and fat). In addition, unlike conventional X-rays, which use a single image projection plan to create a picture, CT creates an image by combining information from numerous tiny projections across the body. Because of this combination of photos, more soft tissue detail may be seen. An axial "slice" is the name of each image in a CT scan [6]. Therefore, CT planes and sliced images should be construed as the object (patient) segmented in an axial plane, with the observer seeing the segment from the bottom to the top. The domains of musculoskeletal and neuroradiology are where MRI is most beneficial. The patient is positioned in an intense field of constant magnetic to get an MRI picture. The magnetic field associates hydrogen nuclei in the patient's body in the magnetic field's direction. An exterior radiofrequency (RF) pulse distracts the nuclei from their existing position alignment. When the RF pulse is halted, the hydrogen nuclei re-align inside the externally imposed magnetic field, emitting RF-indicating signals as the energy deteriorates [5]. The computer examines each RF wave for strength and other factors. The field strength determines the frequency of the RF signal produced by the hydrogen nucleus when it returns to its initial alignment inside the field. As a result, the nucleus's RF signal location may be estimated. The computer then assigns grayscale quantities to signals on the detector. MRI pictures can display different diseases, forming a medical image built on tissue features. It is fundamentally distinct from other body tissues' conventional absorption of X-rays. For example, soft tissue anomalies such as ligament rips, benign tissue cancers, and herniated discs are commonly defined using MRIs, which can discern soft tissue changes better than CT images [7]. The injection of radiopharmaceutical into a patient is the initial step in every imaging procedure done with nuclear medicine diagnostics. A radiopharmaceutical is a radionuclide that has been combined with a pharmaceutical. A radionuclide is an unpredictable radioactive isotope element that generates radiation to sustain its constancy and stability. A nuclear medicine gamma camera can detect this radiation when they become gamma rays. A pharmaceutical substance can be found in healthy or diseased tissue. The physiologic distribution of the medication that is administered determines the nuclear medicine image. A bone scan procedure, for instance, is done by administrating the radiopharmaceutical technetium 99m (Tc-99m) into the human body. Osteoblasts concentrate the medicinal methylene diphosphonate in the mineral stage of bone. Tc-99m is a radionuclide that releases a 140-keV gamma photon that a gamma camera may easily detect [8]. As a result, a bone scan is an illustration that indicates the distribution of active osteoblasts. Breast carcinoma is widespread among women, with more than 200,000 additional cases diagnosed yearly. Understanding the distinction between screening, investigative, and diagnosing mammography is crucial. Early diagnosis and detection of breast cancer by mammography have resulted in a 40% reduction in death. There have also been advancements in breast cancer treatment, using medications that target tumors depending on their biology. Annual mammography examinations are the most effective way to ensure the success of screening mammography. Screening improves the accuracy of identifying minor differences, particularly in denser tissue. The annual mammogram, which detects and photographs the breast tissue in the craniocaudal (CC) and mediolateral oblique view (MLO) perspectives, is screening mammography. After a concerning finding is discovered in screening mammography, diagnostic mammograms are performed to characterize it better. Additional diagnostic images such as views, spot scenes of the lesion in concern, and amplification views are all included in diagnostic mammography [9].

Linking modalities via the picture archiving and communication system (PACS)

PACS is a sophisticated unit utilized to acquire, transmit, store, disseminate, present, and analyze medical images. It has many benefits: transfer speed, productivity, concurrent availability of medical images from remote clinical environments, rapid image evaluation, data storage, and cost-effectiveness. This technique eliminates the requirement for repeated digital image captures and tackles issues such as video damage, the necessity to capture them several times, and the additional costs related to image retakes. Digital imaging and communications in medicine (DICOM) and Health Level 7 (HL7) are communication and integration among modalities and interfaces. PACS, a critical element of modern hospitals' radiology departments and supports the entire institution, costs significant money. Because this system contains many medical data and clinical records, it must be run ideally, managed systematically, and safeguarded from failures and technical alternatives. PACS must also share the vast number of medical data and images it stores with other consumers, such as healthcare professionals from other hospitals and healthcare organizations inside and outside the city and country. This can only be realized if PACS comprises appropriate elements, solutions, and services that can produce ideal functions over a long period [10].

## Review

Method

Relevant literature was reviewed from January 2010 to July 2022 using different scientific databases such as Scopus, PubMed, Web of Science, and ScienceDirect, using different search strategies. Mesh terms used were "Image Processing," "Computer-Assisted," "Image Interpretation," "Computer-Assisted," and "Image Enhancement" and related keywords to image informatics and image in healthcare, and included only English literature.

Results and discussion

The search was conducted and revealed 23 articles that met the search criteria. The results are presented thematically.

The Development of Imaging Informatics

Radiology, pathology, cardiology, endoscopy, and other clinical departments use imaging informatics. There are various aspects of imaging informatics, including:

Structured Reporting

The medical record is built on the foundation of written conversations between physicians. Structured reporting creates communications that use a standardized vocabulary and report schemas that are generally template-based. In radiography, point-and-click methods enable structured reporting that extends over 40 years. The increased adoption of commercial speech-recognition technologies in the last 10-15 years has fueled interest in template-based reporting. The necessity to produce vast numbers of reports in radiology necessitates a system, including a "talking template," which enables doctors to maintain their gaze on the images during digital dictation. Structured reporting is recommended to boost efficiency, increase quality, and enhance data collection. Structured reports, according to recent studies, have upgraded the reporting process in general [11].

Ontologies

An ontology is a human-readable and machine-processable knowledge model that explicitly describes the concepts in a realm of dialogue and their connections. The BioPortal portal of the National Center for Biomedical Ontology (NCBO) provides over 300 ontologies in various biomedical and health-related areas. However, medical imaging is just moderately included in most basic health ontologies. The RadLex ontology has been developed over the last 10 years to fill that need and give a unified language for radiology. RadLex incorporates divisions of important ontologies, such as the foundational model of anatomy, and provides the vocabulary for examination, illnesses, imaging equipment, insights, anatomy, and workflow [12].

Vendor Neutral Archive (VNA)

Because of the necessity to distribute this information across divisions and caregivers, centralized archiving systems with the ability to interface with different view applications and electronic health records were required. This need for communication aided the rise of new solutions. In addition, enterprise-imaging (EI) systems, which substitute departmental information silos with centralized healthcare enterprise databases, were developed in response to the requirement to collect and distribute imaging data from numerous divisions. VNA implementation has been associated with cost savings, storage reduction, and enhanced disaster recovery solutions [13].

Natural Language Processing

Most medical imaging procedures have narrative language in their clinical reports. Several studies in NLP have used imaging reports as materials. NLP research in the 1990s was centered on analyzing, regulating, and mapping phrases to an official standard model to express health and clinical data. Negation-detection algorithms, such as NegEx, have improved the precision with which confident and adverse claims in a narrative-free text can be identified. Extra work has also been done on obtaining suggestions from imaging, reports, reliable disease diagnosis, and clinically relevant incidental findings [14].

Standards and Interoperability

The terms Digital Imaging and Communications in Medicine (DICOM) and Integrating the Healthcare Enterprise (IHE) are commonly used in imaging informatics. DICOM is an international standard for transferring medical picture data. IHE is a cooperation of expert associations that aims to increase the interoperability and exchange of healthcare information technology (IT) systems by identifying preferred implementations of existing standards called "profiles" rather than inventing new ones. Interoperability means the capability of various healthcare IT frameworks to interact with each other and consistently interpret information. For example, the Health Level 7 (HL7) standard communicates textual information between medical apps and equipment. The ACR and the National Electrical Manufacturers Association (NEMA) to develop the Digital Imaging and Communications Standards Committee to produce a voluntary standard to focus on the challenges of retrieving and accessing digital images. The ACR-NEMA 300-1985 Standard established DICOM in 1985. In 1988, ACR-NEMA 300-1988 was released. Several suppliers implemented the standard and tested the implementation's success. Another edition of the standard was created, and completed in 1992, moved to operate via typical computer systems, and altered its name to DICOM to include worldwide partners. Although DICOM began as a standard for radiology, it has expanded to include endoscopy, cardiology, pathology, and radiation therapy. DICOM is an exchange standard for storing and archiving image data. The service-object pair is DICOM's most basic functional unit (SOP). An SOP comprises a service class and an information object definition that has been instantiated (IOD). An IOD can be believed to be a structure with blank fields at the beginning. The fields are assigned values during the instantiation process, changing the primarily empty IOD into a DICOM data set. As a request to save or transport data, an application-level provision is a service class. As a result, the SOP indicates a direct command (service class) that should be utilized for the newly created IOD (DICOM data set) [15]. IHE is sometimes misinterpreted as a known standard, which is different. Instead, IHE uses current standards to assist in integrating a wide range of healthcare IT systems to improve healthcare. It originated as cooperation among the Radiological Society of North America (RSNA), the Healthcare Information and Management Systems Society (HIMSS), numerous university institutions, besides several medical imaging companies to address interface integration difficulties throughout the healthcare informatics spectrum. IHE uses use cases to represent situations that describe workflows/processes involving multiple systems/medical technologies and the interoperability of the communication elements. A use case is a recognized workflow description, including players' insights who trade operations to achieve goals. IHE achieves the aims of real-world use cases using existing standards (i.e., DICOM, HL7) and offers implementation guidance in the manner of integration profiles. In addition, IHE collaborates with other standard bodies to enhance their standards when existing standards need to address a use case. IHE also sponsors yearly global gatherings known as connections. Suppliers converge to determine IHE compliance and demonstrate product interoperability levels [16].

Computer-Aided Detection and Diagnosis

Since the introduction of computers for obtaining and presenting medical images, there has been an interest to know if computers could be utilized to investigate pictures to identify and help detect and diagnose illnesses. The purpose of computer-aided detection (CAD) and computer-aided diagnosis (CADx) is to reduce the likelihood of such errors while increasing overall accuracy and efficiency. Although static computer instructions can be used to create CAD and CADx systems, machine learning (ML) is an innovative algorithmic technique used to make dynamic judgments. The study of artificial intelligence (AI) led to the development of ML. Evaluating simple examinations will become increasingly automated as CAD and CADx algorithms advance. We fully expect simple matters to be assessed by humans only on rare occasions in the future. As a result, radiologists will have more time and energy to attend to the most complex and challenging situations. This will increase diagnostic accuracy and efficiency and improve patient outcomes [17].

Business Analytics and Operational Analysis

Business analytics collects, assesses, and presents data revealing company activities. This word applies to the conventional business data of profits and expenditures and how patients roll through with a facility in the case of a hospital or healthcare organization. For example, what is the patient's waiting time and turnaround time within each department or facility? Is there any downtime? How often does it need to be repaired? Did the equipment lead to downtime? Do we have constant preventative maintenance? Do we have an active contact? Some data may indicate current issues; for example, if no image production was detected in two hours, it could suggest that none has required imaging and that the equipment is down. Business analytics assists practitioner leaders in addressing these issues and making sound judgments and decisions [17, 18].

Imaging and Big Data

The pixel data and the informational text report are considered big data in medical imaging. Due to the massive amount of big data, advanced algorithms are required to mine and evaluate it. In addition, big data analytics can uncover significant connections between a disease's imaging and clinical features. As big data becomes more widely used in healthcare, suppliers and vendors must ensure that the Health Insurance Portability and Accountability Act (HIPAA) guidelines are met and that inaccessible health information is kept secure [19]. In the last 25 years, the knowledge and uses of informatics in medical imaging have improved tremendously. For example, PACS and teleradiology have been made possible by advancements in requirements and technologies for the solidity and communication of digital pictures. In addition, the ability to generate and interpret imaging procedure reports has improved thanks to advances in speech recognition, natural language processing (NLP), structured reporting, and ontologies. Due to the increased volume of medical picture data, informatics has created solutions to address workflow and ergonomic challenges. Computer-assisted detection and diagnosis of anomalies in medical imaging have created new possibilities for bettering patient care. When combined with high-dimensional genetic data, the expanding quantity of medical imaging exams and their massive amounts of data make a perfect stand for "big data" analytics platforms. Radiogenomics examines the link between a disease's genetics and its characteristics; this new discipline can improve our understanding of disease biology, prediction, and therapy alternatives. Shortly, informatics and medical imaging will have unprecedented opportunities to progress in both disciplines and improve health [17-19].

Teleradiology

The requirement for after-hours coverage for urgent and emergent radiologic examinations was historically the critical motivator for teleradiology, where radiologists diagnose medical images remotely and offsite. In addition, some institutions use teleradiology for regular readings. However, the value of teleradiology, and its control, are hotly debated in the radiology profession, especially in instances where local radiology is either nonexistent or poor, and the quality of the remote service is guaranteed by specified credentials and certification standards [20].

Imaging Informatics, Machine Learning, and Artificial Intelligence

ML is an interdisciplinary AI field that depends on computer science, neuroscience, psychology, and statistics breakthroughs to create computer algorithms tasks from data without being explicitly programmed. For example, it can enable computers to recognize trends in data or make predictions depending on previous occurrences. Over the last few decades, medical imaging has become increasingly common in clinical settings. The massive expansion of variable imaging procedures and the latest developments in the ML profession have recently demonstrated even higher potential and applicability. For oncology usage, ML can extract meaningful imaging data. The first two examples correlate radionics using classical analysis with the new deep convolutional neural network (CNN) approach. In contrast, the third example will use deep learning to detect metastatic breast cancer in response to current grand obstacles. Recent ML algorithms are practical tools to enhance medical practice by decreasing human effort and potential errors. Moreover, augmenting human perception will improve patients' diagnostic and treatment accuracy. In general, an exciting digital age in the medical industry is needed, particularly in oncology, thanks to the newest advances in ML techniques. At large, patients, their doctors, and society are projected to benefit from ML since it will allow for more optimal utilization and save time and money on unneeded medical bills [21]. AI is moving towards the top of exaggerated prospects, agreeing to the 2019 Gartner Hype Cycle for developing technologies. In specific ways, radiology AI has a low entrance hurdle. Journal websites publish news pieces and manuscripts revealing novel AI applications in radiography, such as publicly available labeled datasets like those from the National Lung Screening Trial (NLST). Various free outsourced online ML platforms provide pre-packaged algorithms and limited computing capacity, allowing users to train an AI system. Using these resources, any user can immerse in AI imaging and train a model to accomplish a primary recognition task, such as discovering a lung nodule. However, the seeming simplicity of the above explanation betrays the task's actual intricacy. It is conceivable for an AI model to generate a product that appears suitable but is not clinically relevant or valid. Does the data genuinely correspond to what they argue; is the model accuracy of >90% corresponding to high execution? Does a model act as intended on new data? How to overcome overfitting if occurred? With the growing demand for data sharing, radiology practices are suddenly confronted with new data security and privacy challenges. Furthermore, adding new devices to a radiologist's arsenal necessitates that they fit effortlessly into the demanding and frenetic medical workflow without interfering with the operation of existing practices, endangering patients, or reducing the doctor's productivity and efficiency [22]. Additionally, although image processing to detect results has been the emphasis of radiology AI, other data types created in a radiology service could gain from AI technologies, such as reports, workflow phases, and others. In COVID-19 applications, AI offers safe, precise, and effective imaging solutions. The applications analyze intelligent imaging systems, clinical diagnostics, and novel research in-depth, encompassing the complete pipeline of AI-powered imaging applications. To illustrate the efficiency of AI-inspired medical imaging for COVID-19, X-rays and CT are mainly used [23].

## Conclusions

Imaging informatics is crucial to achieving AI application in radiology for all the reasons earlier. An imaging informatician is a distinctive expert who works at the junction of data science, clinical radiology, and information technology (IT). Imaging informaticians are becoming crucial participants in creating, validating, and implementing AI in the clinical setting because they can grasp each area and translate among specialists in the field.
